# Additive genetic variation for tolerance to estrogen pollution in natural populations of Alpine whitefish (*Coregonus* sp., Salmonidae)

**DOI:** 10.1111/eva.12216

**Published:** 2014-10-03

**Authors:** Gregory Brazzola, Nathalie Chèvre, Claus Wedekind

**Affiliations:** 1 Department of Ecology and Evolution, Biophore, University of LausanneLausanne, Switzerland; 2 Institute of Earth Surface Dynamics, University of LausanneLausanne, Switzerland

**Keywords:** 17*α*-ethinylestradiol, embryo development, fluconazole, micropollutants, Salmonidae, timing of hatching

## Abstract

The evolutionary potential of natural populations to adapt to anthropogenic threats critically depends on whether there exists additive genetic variation for tolerance to the threat. A major problem for water-dwelling organisms is chemical pollution, and among the most common pollutants is 17*α*-ethinylestradiol (EE2), the synthetic estrogen that is used in oral contraceptives and that can affect fish at various developmental stages, including embryogenesis. We tested whether there is variation in the tolerance to EE2 within Alpine whitefish. We sampled spawners from two species of different lakes, bred them *in vitro* in a full-factorial design each, and studied growth and mortality of embryos. Exposure to EE2 turned out to be toxic in all concentrations we tested (≥1 ng/L). It reduced embryo viability and slowed down embryogenesis. We found significant additive genetic variation in EE2-induced mortality in both species, that is, genotypes differed in their tolerance to estrogen pollution. We also found maternal effects on embryo development to be influenced by EE2, that is, some maternal sib groups were more susceptible to EE2 than others. In conclusion, the toxic effects of EE2 were strong, but both species demonstrated the kind of additive genetic variation that is necessary for an evolutionary response to this type of pollution.

## Introduction

One major question in conservation biology is whether natural populations can adapt early enough to the various anthropogenic challenges they are exposed to before they go extinct (Ferrière et al. 2004; Hendry et al. 2011). Among the major challenges that water-dwelling organisms have been newly exposed to during the last decades are various sorts of chemical pollution through residues in effluents of sewage treatment plants. Among the most common pharmaceuticals that enter the environment after passing municipal sewage treatment and that have well been identified as aquatic environmental risk are the natural steroid estrogen hormone estrone (E1), 17*β*-estradiol (E2), and 17*α*-ethinylestradiol (EE2) (Caldwell et al. 2012). The latter (EE2) is used in most formulations of oral contraceptive pills because it mimics the endogenous hormone E2 and is more stable than its natural counterpart (Kime 1998). In the aquatic environment, EE2 is also more persistent than natural estrogens (its half-life is about 14 days, Shore et al. 1993). EE2 is now commonly found in surface waters at concentrations around 1 ng/L (e.g., Larsson et al. 1999; Vulliet and Cren-Olive 2011; Zhang et al. 2011), but concentrations of 17.2 ng/L (Beck et al. 2005), 42 ng/L (Ternes et al. 1999), and up to 831 ng/L (Kolpin et al. 2002) have been reported, and concentrations of >1 ng/L are sometimes even found in groundwater (Vulliet and Cren-Olive 2011).

EE2 is a potent endocrine disruptor in fish (Kime 1998; Gutendorf and Westendorf 2001; Lange et al. 2001) and has been shown to influence viability and development of zebra fish embryos (*Danio rerio*), either directly as immediate response to an exposure or indirectly via the effects of parents that had been exposure to EE2 (Soares et al. 2009). Overall, the studies so far suggest that embryos are more susceptible to the immediate toxic effects of EE2, while later life history stages may suffer more from the effects EE2 has on sex determination and reproduction (e.g., Segner et al. 2003a; Soares et al. 2009; Harris et al. 2011). Concentrations around 1 ng/L can induce vitellogenin production in male rainbow trout (*Oncorhynchus mykiss*) and zebra fish (Rose et al. 2002) and significantly reduce fertilization success (Segner et al. 2003b). Higher concentrations are known to affect reproductive behavior or sexual characteristics or lead to intersex in, for example, zebra fish (Larsen et al. 2008), fathead minnow (*Pimephales promelas*) (Lange et al. 2001), three-spined sticklebacks (*Gasterosteus aculeatus*) (Dzieweczynski 2011), or the whitefish *Coregonus lavaretus* (Kipfer et al. 2009). Moreover, exposure to substances with as high an estrogenic potency as EE2 is expected to influence sexual differentiation in fish where sex is genetically determined but can be reversed by environmental factors which is the case in many fishes of various families (Devlin and Nagahama 2002; Stelkens and Wedekind 2010). EE2 could be demonstrated to arrest male differentiation in zebra fish when applied during the period of sexual differentiation (Van den Belt et al. 2003; Fenske et al. 2005). Sex ratio management via exposure to hormones is therefore widely used in aquaculture (e.g., if one sex is preferred for economic reasons) (Baroiller et al. 2009) and has been discussed in the context of conservation management (Wedekind 2002b, 2012; Gutierrez and Teem 2006). Estrogens as pollutants in effluents of sewage treatment plants are therefore likely to induce sex reversal and sex ratio distortion in wild fish populations (Jobling et al. 2006; Scholz and Kluver 2009). Indeed, a field experiment on roach (*Rutilus rutilus*) resulted in 98% phenotypic females after 3.5 years of chronic exposure to treated estrogenic wastewater effluents and still 79% phenotypic females in a 50% dilution of these effluents (Lange et al. 2011). On the long term, a biased sex ratio is a serious threat to natural populations because it can considerably reduce genetically effective population sizes, drive sex chromosomes to extinction, and may affect sex ratios in some counterintuitive ways (Cotton and Wedekind 2009). However, Hamilton et al. (2014) recently found populations of roach (*R. rutilus*) to be self-sustaining in heavily estrogen-polluted waters and despite widespread feminization. Such observations raise the question whether natural populations can adapt in useful time to this rather new type of pollution, that is, whether there can be rapid evolution in response to the pollution (Wedekind 2014).

Despite the possible relevance of estrogen pollution worldwide, it is still unclear whether rapid evolutionary changes are possible within natural populations in response to the potential negative effects that estrogens such as EE2 may have on average viability and growth in natural fish populations. First, it needs to be established whether there is, under controlled conditions, phenotypic variation in response to this selection pressure. It would then be necessary to understand the nature of such phenotypic variation, that is, whether it is due to genetic differences, individual phenotypic plasticity, maternal environmental effects, epigenetic factors, or any form of nongenetic inheritance (Bonduriansky and Day 2009; Hendry et al. 2011; Vandegehuchte and Janssen 2011).

Here, we sampled two natural whitefish populations (*Coregonus* sp.) to (i) study the toxicity of EE2 to embryos and (ii) test whether there is the kind of phenotypic and genetic variation within populations that would be necessary for a rapid evolutionary response to this type of pollution. Alpine whitefish are plankton feeders and typically keystone species in the larger lakes of the pre-Alpine region. The two whitefish species we chose differ in many respect and may hence cover much of the diversity within the Alpine whitefish species complex: a fast-growing, large-type whitefish from the Lake Geneva (*Coregonus palaea* Fatio 1890) and a slow-growing, small-type whitefish from the Lake Brienz (*Coregonus albellus* Fatio 1890). The two lakes are about 100 km apart and belong to different drainage systems. While Lake Brienz has been described as ‘ultra-oligotrophic’ (Müller et al. 2007) and can be assumed to be comparatively weakly exposed to municipal effluents (few small communities in the catchment area), the state of eutrophication of Lake Geneva has been ranked as moderate to strong (Vonlanthen et al. 2012), and the spawning place of the *C. palaea* study population is close to city of Lausanne (with >300 000 inhabitants living in the city and its agglomeration), that is, exposure to municipal effluents can be assumed in the upper range within Switzerland. We sampled adult breeders from their spawning sites, used their gametes to produce all possible half-sib groups, and exposed the resulting embryos singly to one of several concentrations of EE2 to study growth and survival until hatching. Full-factorial *in vitro* breeding allowed us to separate additive genetic from maternal environmental effects (variation in egg quality) on the susceptibility or tolerance of embryos to estrogen pollution (Lynch and Walsh 1998; Wedekind et al. 2007b).

## Methods

### Sampling and experimental treatment of *Coregonus palaea*

Adult large-type whitefish (‘Palée’; *C. palaea*) from Lake Geneva, Switzerland, were caught with gill nets during their breeding season in December. Four females and six males were stripped to collect their gametes for *in vitro* fertilizations in a full-factorial breeding design. For this, the eggs of each female were distributed to six new petri dishes in about equal amounts, and milt was added and activated with few millilitre of water to produce all 24 possible half-sib groups. The freshly fertilized eggs were left undisturbed for at least 1 h to allow for egg hardening. They were then transported to the laboratory were they were immediately washed as in von Siebenthal et al. (2009). In total, 2304 eggs (96 eggs per sib group) were then distributed singly to 24-well cell culture plates (Falcon; Becton Dickinson, Allschwil, Switzerland; 2 mL wells). The wells contained 0, 1, 10, and 100 ng/L of analytical 17α-ethinylestradiol (Sigma-Aldrich, Buchs, Switzerland). All water used here had been chemically standardized, that is, reconstituted according to the OECD guideline No. 203, Annex 2 (OECD 1992), tempered, and aerated before use. The embryos were incubated at constant 6.5°C. Embryo mortality and the timing of hatching were recorded daily from day 13 onward (the last dead embryo was recorded at day 123 after fertilization). *Coregonus* sp. eggs are much more translucent than, for example, typical *Salmo* sp. or *Oncorhynchus* sp. eggs, and embryos are easily recognizable after few days of incubation, but it remains difficult to distinguish dead embryos from unfertilized eggs during the very first days of incubation (because they both turn white after rupture of the yolk membrane; Leitritz and Lewis 1976). Therefore, the first recording of mortality at days 13 could include unfertilized eggs and was therefore separately analyzed and interpreted. Permissions for sampling adults, *in vitro* breeding, and the raising of embryos in the laboratory were granted by the fishery inspectorate of the Vaud canton.

### Sampling and experimental treatment of *Coregonus albellus*

Adult small-type whitefish (‘Brienzlig’; *C. albellus*) from Lake Brienz, Switzerland, were caught with gill nets during their breeding season in September. To minimize temperature variation (the fish spawn in about 60–80 m depth at about 5°C), the fish were immediately transported in cold water to a refrigerated van (IVECO 3T5) where gamete collection and *in vitro* fertilization were done at 5°C as described above. Four females and five males were stripped and used to produce all possible sib groups. After egg hardening, the freshly fertilized eggs were transported to the laboratory and washed, and in total, 1600 eggs (160 per sib group) were distributed to 24-well plates as described above. They were exposed to 0, 1, 5, 10, 50 or 100 ng/L analytical 17α-ethinylestradiol (Sigma-Aldrich).

Whitefish from Lake Brienz show very low growth rates and body condition as compared to other Alpine whitefish (probably because Lake Brienz is an ultra-oligotrophic lake; Müller et al. 2007) and female *C. albellus* produce comparatively few and small eggs (Kirchhofer and Lindt-Kirchhofer 1998) that may be less resistant to handling as other Alpine whitefish. We therefore ran two further controls treated with antimicrobials to potentially reduce stress-induced embryo mortality in the laboratory (Wedekind et al. 2010), additional to the 0 ng/L EE2 control. These further controls were treated with 10 or 100 ng/L analytical fluconazole (Sigma-Aldrich), a broad-spectrum antifungal drug. We did not combine the antimicrobial and the EE2 treatments. The antimicrobial treatment in the additional controls was solely to learn more about the potential relevance of microbes for embryo mortality of a species that is expected to be difficult to raise under experimental conditions.

Embryo mortality and hatching were recorded daily from day 16 postfertilization onward. As in the upper experiment with *C. palaea*, the first recordings of mortality at day 16 could include unfertilized eggs and were therefore separately analyzed and interpreted. Incubation temperature was planned to be constant at 6°C, but because of technical problems went up to 15°C for few hours at day 10 and again at day 14 postfertilization. Hatchlings (alevins) were photographed (Olympus C-5060; Olympus, Shinjuku, Japan) in a drop of water under a microscope on the first and the tenth day after hatching. The notochord length and the volume of the yolk sac of individual hatchlings were determined from these photographs using the open-access software imagej 1.42q (http://imagej.nih.gov/ij/). Developmental time was determined as degree days (dd). All measurements were taken blindly with respect to the experimental treatment. The expected notochord length at the time the yolk sac would be used up was linearly extrapolated from loss of yolk sac volume and increase of alevin length during the first 10 days. Permissions for sampling adults, *in vitro* breeding, and the raising of embryos in the laboratory were granted by the fishery inspectorate of the Bern canton.

### Statistics

Within each experiment, the exposure to estrogen concentrations was full factorial and balanced with respect to parental origin. Parental effects, main effects of EE2 treatment, and treatment × parent interactions were tested either in generalized linear models (on embryo survival) or three-way anovas on continuous dependent variables such as alevin size and growth. All analyses were based on embryo as independent replicates, with treatment and parental origin as fixed factors (we refrained from including second-order interaction terms and from estimating average sire or dam effects because of limited sample sizes per populations). Two male *C. albellus* were excluded from all analyses because total mortality of their offspring turned out to be 100% and 99.2%, respectively. Main treatment effects were tested in directed heterogeneity tests (Rice and Gaines 1994) based on the expectancy that if estrogens have an effect on embryo survival and life history, the effects would increase with increasing estrogen concentrations. Data were analyzed in jmp 9.0 (SAS Institute Inc., Cary, NC, USA) and r 2.14.1 (R Development Core Team 2011).

## Results

### Embryo mortality

**Figure 1:**
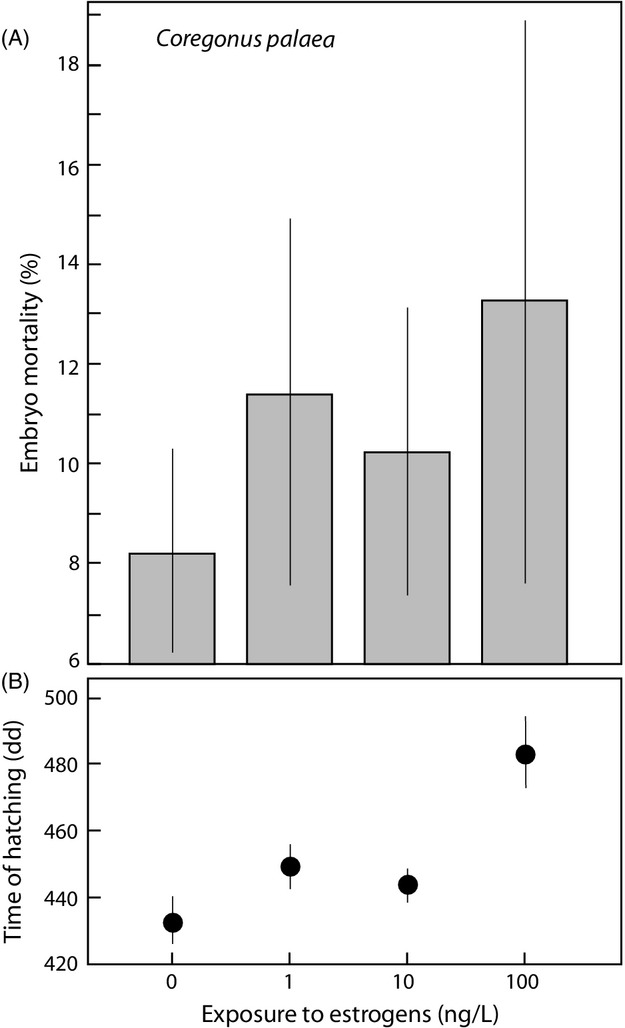
Experiments on *Coregonus palaea*: effects of exposure to the estrogen EE2 on (A) embryo mortality and (B) average timing of hatching (in degree days). The panels show means and the 95% confidence intervals based on family means. See text for statistics.

**Figure 2:**
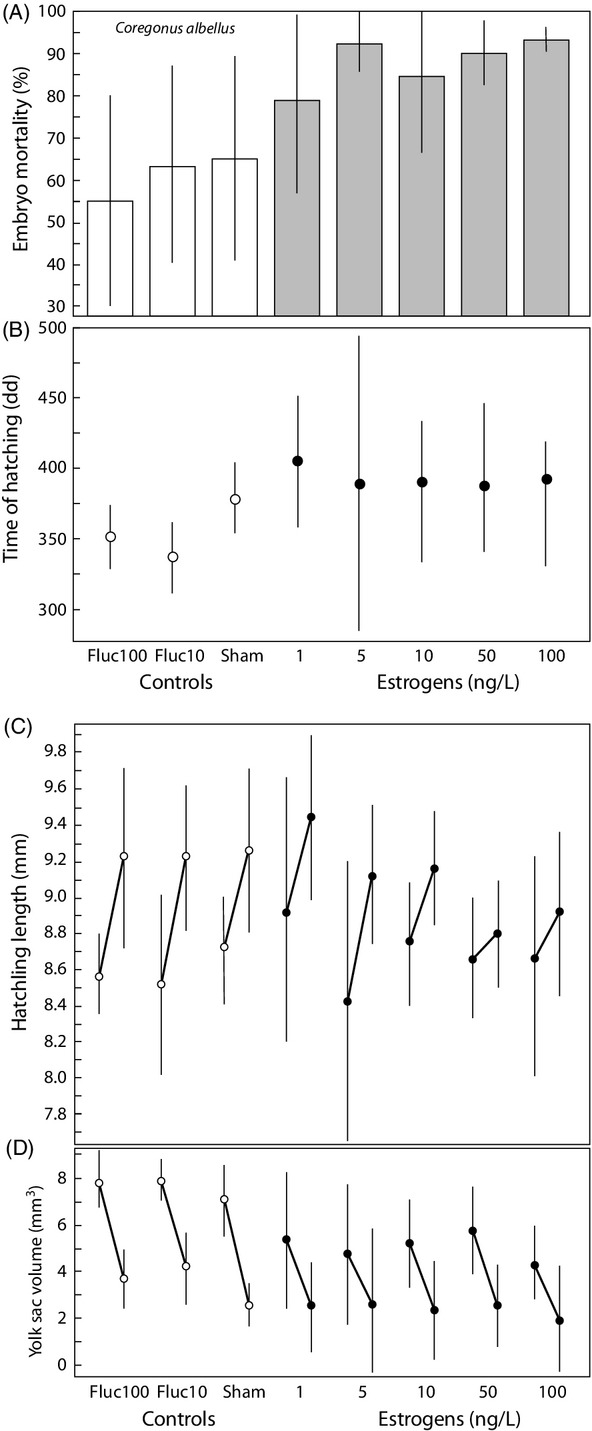
Effects of different experimental stress treatments on embryo mortality, timing of hatching, and hatchling growth in *Coregonus albellus*. Embryos were either treated with 100 ng/L (‘Fluc100’) or with 10 ng/L fluconazole (‘Fluc10’) to reduce microbial stress, sham treated, or exposed to various concentrations of estrogens. (A) Embryo mortality, (B) timing of hatching of the survivors (in degree days), (C) hatchling length one day and 10 days after hatching, (D) yolk sac volume one day and 10 days after hatching. All panels show means and the 95% confidence intervals based on family means. See text for statistics.

Increased exposure to estrogens increased embryo mortality until hatching in *C. palaea* (*χ*^2^ = 7.5, df = 3, r_s_P_c_ = 0.75, *P* < 0.05; Fig.1A) and in *C. albellus* (*χ*^2^ = 52.1, df = 5, r_s_P_c_ = 0.75, *P* < 0.01; Fig.2A). The fact that very early dead embryos are difficult to distinguish from nonfertilized eggs did not seem to play a role here, because the respective tests on the earliest recording of mortality, that is, the only mortality recording that could include unfertilized eggs revealed no significant treatment effects (*C. palaea*, day 13: *χ*^2^ = 3.5, r_s_P_c_ = 0.14, *P* > 0.05; *C. albellus*, day 16: *χ*^2^ = 5.1, r_s_P_c_ = 0.43, *P* > 0.05). Models that include EE2 treatment, dam, and sire effects revealed additive genetic variance for tolerance to EE2 in both whitefish species (the significant treatment × sire effects in Table1), additionally to the overall additive genetic variance in viability that we found in both species (the significant sire main effects in Table1), and the nonadditive genetic variance in viability that we found in *C. palaea* (the significant dam × sire effect in Table1a).

**Table 1:** Effect likelihood ratio tests on embryo mortality until hatching in (a) *Coregonus palaea* from Lake Geneva and (b) *Coregonus albellus* from Lake Brienz treated with various concentrations of the synthetic estrogens EE2.

Factor	*χ*^2^	df	*P*
(a) *C. palaea* (*N*_total_ = 2304)			
Treatment (T)	3.9	3	0.27
Dam (D)	6.9	3	0.08
Sire (S)	28.8	5	<0.0001
T × D	13.3	9	**0.15**
T × S	25.6	15	**0.04**
D × S	25.1	15	0.05
(b) *C. albellus* excluding extra controls (*N*_total_ = 719)			
Treatment	37.9	5	<0.0001
Dam	7.3	1	0.007
Sire	19.5	2	<0.0001
T × D	12.6	5	**0.03**
T × S	21.0	10	**0.02**
D × S	0.05	2	0.98

*P*-values linked to parent × treatment effects are emphasized in bold.

### Timing of hatching

We found significant dam and sire effects on the timing of hatching in both species (the main effects in Table2). Estrogen treatment had a delaying effect on the timing of hatching in *C. palaea* (*F*_3,2054_ = 167.7, r_s_P_c_ = 0.80, *P* < 0.01; Fig.1B). This could be confirmed in a three-way anova that included the parental effects (treatment main effect in Table2a). This anova revealed additive genetic variance for the timing of hatching in response to the estrogen exposure (the significant treatment × sire effect in Table2a). We also found significant a treatment × dam effect (Table2a) and significant nonadditive genetic variance in response to the estrogen treatment (the dam × sire effect in Table2a). None of these effects of EE2 treatment on the timing of hatching could be confirmed in *C. albellus*: Neither was the timing of hatching increasingly delayed with increasing estrogen concentration (*F*_5,110_ = 1.3, r_s_P_c_ = 0.19, *P* > 0.05; Fig.2B), nor was there any significant parental effect in reaction to the treatment (Table2b). However, if the two additional controls that were treated with antimicrobials were included into the models, hatching was delayed with increased stress level (*F*_5,110_ = 1.3, r_s_P_c_ = 0.19, *P* < 0.05; Fig.2B; the treatment effect in a three-way anova analogous to the one in Table2b would be: *F* = 7.2, df = 6, *P* < 0.0001).

**Table 2:** anova on the timing of hatching (a) in *Coregonus palaea* and (b) in *Coregonus albellus* (notation as in Table1). In (b), some degrees of freedom were lost because of high mortality in some experimental cells.

Factor	*F*	df	*P*
(a) *C. palaea* (*N*_total_ = 2055)			
Treatment	239.0	3	<0.0001
Dam	64.8	3	<0.0001
Sire	38.7	5	<0.0001
T × D	11.6	9	**<0.0001**
T × S	6.2	15	**<0.0001**
D × S	4.2	15	<0.0001
(b) *C. albellus* excluding extra controls (*N*_total_ = 115)			
Treatment	0.4	4	0.80
Dam	19.3	1	<0.0001
Sire	4.7	1	0.03
T × D	1.9	5	**0.11**
T × S	0.7	9	**0.67**
D × S	2.3	2	0.11

*P*-values linked to parent × treatment effects are emphasized in bold.

### Alevin size and growth

The body length of freshly hatched *C. albellus* alevins did not seem to be affected by the estrogen treatment (*F*_5,109_ = 0.88, r_s_P_c_ = 0.16, *P* > 0.05; Fig.2C). However, yolk sac volume at the time of hatching was reduced (*F*_5,109_ = 2.0, r_s_P_c_ = 0.71, *P* < 0.01; Fig.2D). Accordingly, negative effects of estrogen on hatchling length could be recorded 10 days after hatching (*F*_5,109_ = 4.4, r_s_P_c_ = 0.88, *P* < 0.001; Fig.2C) and at the expected final alevin size (*F*_5,109_ = 4.3, r_s_P_c_ = 0.89, *P* < 0.001). Hatchling growth was not only reduced during the first 10 days after hatching (*F*_5,109_ = 3.0, r_s_P_c_ = 0.59, *P* < 0.05; Fig.2C), but also the potential for further growth as yolk sac volume after 10 days was smaller with increasing exposure to estrogens (*F*_5,109_ = 1.0, r_s_P_c_ = 0.49, *P* < 0.05; Fig.2D).

In *C. albellus*, alevin size at hatching was dependent on maternal effects (the significant dam effects in Table3a), and even if no sire effects could be found on alevin size on hatching or later (Table3), there was still significant additive genetic variance on growth because the time at which final alevin size was reached depended not only on dam but also on sire effects (Table4). Estrogen treatment affects alevin growth differently for different dams (the treatment × dam effects in Table3b,c).

**Table 3:** anova on *Coregonus albellus* alevin size measured (a) 1 day after hatching, (b) 10 days after hatching, and (c) expected size at the time the yolk sac would be used up (extrapolated from loss of yolk sac volume and increase of alevin length during the first 10 days). Only estrogen- and sham-treated groups are included here. *N*_total_ = 114 for each statistical model. Including the two extra controls (the antimicrobial treatments) would not change any conclusions except that the main dam effects would always be significant at *P* < 0.001.

Factor	*F*	df	*P*
(a) *C. albellus* 1 day posthatching			
Treatment	0.06	4	0.99
Dam	14.2	1	0.0003
Sire	0.05	1	0.82
T × D	0.1	5	**0.99**
T × S	1.3	9	**0.23**
D × S	1.2	2	0.30
(b) *C. albellus* 10 day posthatching			
Treatment	2.1	4	0.08
Dam	2.7	1	0.10
Sire	1.7	1	0.19
T × D	2.6	5	**0.03**
T × S	0.7	9	**0.73**
D × S	0.8	2	0.43
(c) *C. albellus* expected final alevin size			
Treatment	2.1	4	0.09
Dam	3.0	1	0.09
Sire	1.9	1	0.18
T × D	2.5	5	**0.04**
T × S	0.7	9	**0.71**
D × S	0.9	2	0.43

*P*-values linked to parent × treatment effects are emphasized in bold.

**Table 4:** anova on the total duration of embryo and larval development in *Coregonus albellus*, extrapolated from the yolk sac volume and its reduction during the first 10 days. Only estrogen- and sham-treated groups are included here (*N*_total_ = 114). Including the two extra controls would lead to very similar values and would not change the conclusions.

Factor	*F*	df	*P*
Treatment	0.1	4	0.96
Dam	10.4	1	0.002
Sire	3.9	1	0.05
T × D	1.3	5	**0.25**
T × S	0.7	9	**0.73**
D × S	1.2	2	0.32

*P*-values linked to parent × treatment effects are emphasized in bold.

## Discussion

Estrogen pollution is a threat to the aquatic environments that has raised much concern (Sumpter 2005; Sumpter and Jobling 2013). Estrogens have repeatedly been demonstrated to induce negative effects on viability and development of fish within various orders (reviews in Scholz and Kluver 2009; Leet et al. 2011; Senior and Nakagawa 2013) and at concentrations that are often found in surface waters (Sumpter and Jobling 2013). We exposed singly kept whitefish embryos to the synthetic estrogen EE2 and found that EE2 significantly reduced viability and growth during embryogenesis even at the lowest concentration of 1 ng/L. Whitefish can therefore be added to the list of ray-finned fish that are very susceptible to estrogen pollution (Scholz and Kluver 2009; Senior and Nakagawa 2013). Increased concentrations of EE2 generally increased embryo mortality in both whitefish species we tested. However, there was much variation in general viability and the susceptibility to EE2 among the different sib groups in our study.

We found significant paternal effects on embryo survival in both species. Paternal origin also had significant effects on development rate in *C. albellus* where we determined embryo growth. Because whitefish are external fertilizers with no parental care, fathers only contribute genes to their offspring, and paternal effects on embryo survival and development rate therefore directly reveal additive genetic variance for general viability within the both populations that we sampled. It turned out that some males were of higher overall genetic quality than others, as previously observed in other samples of Alpine whitefish (Wedekind et al. 2001, 2007a, 2008a; Clark et al. 2014) and other salmonid populations (Jacob et al. 2007, 2010; Pitcher and Neff 2007; Wedekind et al. 2008b; Evans et al. 2010; Clark et al. 2013). Importantly, we also found significant interactions between paternal origin and the EE2 treatment on embryo viability in both species. Such interaction terms demonstrate that some genotypes are more tolerant to EE2 than others, even after controlling for the variation in overall genetic quality within the populations. We conclude that there is, in both study populations, significant genetic variation that would be required for rapid evolutionary responses to EE2 pollution.

When we tested for possible effects of EE2 on embryo growth and development, we found that hatching time was significantly affected in *C. palaea* but not in *C. albellus*. The apparent difference between the two species could be due to differences in sample sizes and the associated statistical power (these differences in sample sizes were partly due to higher embryo mortalities in *C. albellus* than in *C. palaea*; see Methods). In *C. palaea*, we also found hatching time to be generally determined by dam, sire, and dam × sire effects, that is, offspring of half-sib families hatched at different times even if each embryo was raised in isolation. With regard to hatching time, different maternal and paternal half-sib groups also reacted differently to the EE2 treatment. The significant sire × EE2 treatment effect demonstrates again a genetic variation in response to EE2.

Variation in hatching time may either reveal variation in developmental rate (if, at the conditions of our study, hatching is directly linked to a developmental stage) or could reveal a behavioral response to acute stress. Stress-induced precocious hatching is common in amphibians (Warkentin 2011) and has been demonstrated in whitefish in response to waterborne cues of infections or other threats (Wedekind 2002a; Wedekind and Müller 2005). However, in our samples, hatching was generally delayed in EE2-treated embryos. This suggests that the variation in hatching time that we observed revealed variation in developmental rates (as in Clark et al. 2014). The late hatching in EE2-treated embryos therefore suggests that EE2 reduces developmental rates in *C. palaea* and differently so for different genotypes, that is, some genotypes seemed again more susceptible than others to EE2 pollution.

When we analyzed body length and yolk sac volume in *C. albellus* hatchlings, we found not only significant dam effects (some females produced offspring that generally developed faster than those of other females) but also a significant interaction between dam effects and EE2 concentration on embryo growth and expected final size, that is, the progeny of some mothers were more susceptible to EE2 pollution than the progeny of others. Dam effects are expected to be a combination of maternal environmental effects (egg content and egg size) and additive genetic effects. The relative role of the latter remains unclear in this case, because the respective interaction between paternal effects and EE2 concentrations was not significant. However, individual growth rates can be fitness relevant in salmonids (e.g., Skoglund et al. 2012). Therefore, the reduction of embryo growth within some maternal sib groups let us to conclude that there are nonlethal toxic effects of EE2 that may affect fitness among the surviving embryos.

There are a number of differences between the controlled laboratory conditions and natural situations that could potentially affect the toxicity of EE2 and its congeners. Among the micro-ecological factors that could play a role are the composition and density of microbial symbiont communities associated to the embryos (L. G. E. Wilkins, A. Rogivue, L. Fumagalli and C. Wedekind, unpublished data). Very little is currently known about the importance of degradation of estrogenic chemicals in different aquatic environments, that is, it is still difficult to predict environmental concentrations of estrogenic compounds at different times and locations (Sumpter and Jobling 2013). Moreover, it remains to be shown how the effects that different hormone-active chemicals can have on fish development interact, for example, whether and to what degree their toxicity is additive (Sumpter and Jobling 2013). While laboratory studies like ours allow for qualitative conclusions about the existence of genetic and maternal environmental effects (Lynch and Walsh 1998), the relevant quantitative effects of EE2 on embryo growth and development remain to be confirmed under more natural conditions. Basing experiments like ours on larger number of breeders cannot solve this problem, even if larger samples would allow for better estimates of the variance components under our laboratory conditions (as, for example, in Clark et al. 2014).

Since the discovery of Purdom et al. (1994) that estrogenic chemicals in effluents of sewage treatment plants can cause significant alterations in fish, the industry and policy organizations of many countries have significantly invested into the treatment of wastewater to better remove estrogenic chemicals (e.g., Burkhardt-Holm et al. 2008; Sumpter and Jobling 2013). However, while the use of nonylphenol and related chemicals (a group of estrogenic pollutants) could be regulated via legislation in some parts of the world (Sumpter and Jobling 2013), EE2 may be more difficult to ban because it is an active ingredient of most hormonal contraceptives. To the best of our knowledge, no environmental quality standard has yet been defined by any legislative authority. Sumpter and Jobling (2013) suggested that an environmental quality standard of around 0.02 ng/L may be possible, but the authors stressed that the risks of potent chemicals like EE2 should never be fully dismissed even at very low concentrations.

Some whitefish populations in pre-Alpine lakes showed extraordinary high prevalences of gonadal deformations during recent years (Bernet et al. 2009). Potential pollution by endocrine disruptors has been a focus of various studies (e.g., Liedtke et al. 2009; Bogdal et al. 2010). Even if no suspicious contamination levels could be demonstrated so far, all pre-Alpine lakes receive effluents from sewage plants, that is, pollution by EE2 and other estrogens is an environmental risk also in low populated areas. We found that even low concentrations of EE2 would create strong selection pressures on two whitefish species that differ in many respects. Whitefish females produce large numbers of offspring (up to several thousands per year in the case of *C. palaea* and up to several hundreds per year in the case of *C. albellus*). These high reproductive rates in combination with the strong effects EE2 has on embryo survival and growth and the fact that both populations show additive genetic variation in the tolerance to EE2 suggest that rapid evolution in response to endocrine pollution is possible in Alpine whitefish. Our findings further illustrate the importance of genetic variation for natural populations that need to adapt to anthropogenic threats.

### Data archiving statement

Data available from the Dryad Digital Repository: http://doi.org/10.5061/dryad.md103
